# Integrated Biorefinery for a Next-Generation Methanization Process Focusing on Volatile Fatty Acid Valorization: A Critical Review

**DOI:** 10.3390/molecules29112477

**Published:** 2024-05-24

**Authors:** Mohamed Koubaa

**Affiliations:** Université de Technologie de Compiègne, ESCOM, TIMR (Integrated Transformations of Renewable Matter), Centre de Recherche Royallieu—CS 60319, 60203 Compiègne Cedex, France; m.koubaa@escom.fr; Tel.: +33-3-44-23-88-41

**Keywords:** waste stream, anaerobic digestion, methanization, volatile fatty acids, high-value-added products, waste recycling, next-generation biorefinery

## Abstract

This review addresses the critical issue of a rapidly increasing worldwide waste stream and the need for sustainable management. The paper proposes an integrated transformation toward a next-generation methanization process, which leads not only to treating waste but also to converting it into higher value compounds and greener energy. Although the current and commonly used anaerobic digestion process is useful for biogas production, it presents limitations of resource exploitation and some negative environmental impacts. Focusing on the acidogenic stage in waste stream processing, the paper discusses the recent strategies to enhance the recovery of volatile fatty acids (VFAs). These acids serve as precursors for synthesizing a variety of biochemicals and biofuels, offering higher value products than solely energy recovery and soil fertilizers. Additionally, the importance of recycling the fermentation residues back into the biorefinery process is highlighted. This recycling not only generates additional VFAs but also contributes to generating clean energy, thereby enhancing the overall sustainability and efficiency of the waste management system. Moreover, the review discusses the necessity to integrate life cycle assessment (LCA) and techno-economic analysis (TEA) to evaluate the environmental impacts, sustainability, and processing costs of the proposed biorefinery.

## 1. Introduction

The global increase in waste generation by human activity (e.g., industrial, agricultural, etc.), along with the high energy demand and the environmental issues associated with both waste disposal and fossil energy exploitation, has driven the need for innovative and sustainable solutions for the management of waste streams [1]. The valorization of these streams is showing an increased research interest, as it addresses many of the United Nations Sustainable Development Goals. Among the possible solutions, an integrated biorefinery approach seems to be the most suitable in terms of environmental sustainability and economic viability. This approach has the ultimate goal of generating high-value-added products and clean energy from these waste streams with a minimal impact on the carbon footprint [2].

Environmentally friendly technologies have demonstrated significant potential for the sustainable valorization of waste streams via intensification of material fractionation and conversion. These technologies include pulsed electric fields, ultrasounds, and microwaves, among others [3,4,5]. Commonly used thermochemical processes, such as pyrolysis, gasification, and catalytic methods, convert waste into valuable products and energy. Research on pyrolysis and gasification has focused on optimizing the process parameters (e.g., temperature, pressure, residence time) to maximize the yields of bio-oil, syngas, and biochar [6,7]. Additionally, eco-friendly technologies like microwaves and plasma have enhanced the overall efficiency and output [8,9,10,11,12]. Combining these processes with catalytic methods, such as hydrothermal liquefaction and Fischer–Tropsch synthesis, produces diverse and valuable products, including synthetic fuels and chemicals [13,14]. Innovations aim to reduce emissions, improve energy efficiency, and utilize by-products like biochar for carbon sequestration, promoting environmental sustainability. These technologies have been developed to be more scalable and economically viable for industrial applications through improvements in reactor design, material handling, and energy integration.

In addition to the above-mentioned processes, anaerobic digestion, also known as methanization, is one of the most widely applied technologies for biowaste utilization. It involves four main sequential steps: hydrolysis, acidogenesis, acetogenesis, and methanogenesis. These steps are usually grouped into two major phases: hydrolysis/acidogenesis and acetogenesis/methanogenesis [15,16]. Although presenting many scientific and technological gaps, this process is relatively well known, showing an increasing number of methanization facilities around the world at both industrial and agricultural levels. For instance, according to ADEME, the French agency for ecological transition, more than 1450 methanization facilities were running in France in January 2023, with 108 representing historical industrial installations (e.g., agri-food, paper mills, and chemical industries) [17]. These industrial facilities were first installed to treat the organic waste streams generated by the corresponding activity, but the energy crisis of 2022 showed the importance of energy security and autonomy of these companies. The treatment of urban sewage also constitutes a significant sector in France, with 95 facilities focusing on depollution of sewage sludge and a reduction in its volume. Recently, an energy optimization of these facilities was conducted through the injection of biomethane into the gas network (35 facilities among the 95 in 2022 compared to only one unit in 2015). Farm facilities also represent a significant number, with 1238 among the 1450 cited above. In total, ≈100–150 new facilities have been installed annually in France over the last three years [17].

While anaerobic digestion is effective in reducing greenhouse gas emissions, treating biowaste streams, and generating biogas and biofertilizers, the overall process could be optimized for better efficiency. The biogas produced is of relatively low value, and the costs associated with pre-treatment reduce the overall cost effectiveness [18]. Additionally, the impact of biofertilizers on soil biology remains unclear and may present some negative issues [19]. Furthermore, the current technological development is rather empirical and rarely relies on a fine understanding of biological, chemical, and physical phenomena. Reactional phase decoupling allows the study of methanization phases (i.e., hydrolysis, acidogenesis, acetogenesis, and methanogenesis) separately, optimizing and understanding the mechanisms involved in each stage, along with the antagonistic and/or synergistic effects. For instance, it has been demonstrated that a two-stage anaerobic digestion increased the energy recovery from biomass compared to that of one-stage digestion [20,21].

A wider overview of the methanization process could lead to better efficiency, keeping in mind that an integrated biorefinery may generate high-value-added products through the intermediates (e.g., volatile fatty acids, VFAs) formed during the methanization stages (e.g., acidogenesis) [22], through the conversion of gases (e.g., CO_2_ and CH_4_) by further thermochemical processes (e.g., reforming [23,24]), or through extraction of valuable compounds from the digestate before soil-spreading [25].

A special focus of this review will be on the intermediates formed during the acidogenesis stage, particularly VFAs. These molecules, including acetate, propionate, and butyrate, are crucial intermediates in the synthesis of numerous valuable compounds and serve as primary substrates for various biotechnological applications [26,27]. Over the last decade, high research interest has been observed in the production and application of these VFAs from different waste streams [28,29,30,31]. Furthermore, the residues generated from VFA processing, including exhausted media and microbial biomass, present additional feedstocks that can be further converted into biogas (i.e., CH_4_ and H_2_) using either biological or thermochemical processes (e.g., gasification). Such strategies not only favor a better energy valorization process but also follow the principles of the circular economy that encourages the intensification of resource exploitation.

A successfully integrated biorefinery scenario requires deep scientific and technological investigations, including more efficient pre-treatments to enhance biomass hydrolysis, a better understanding of the microbial consortia at each stage of the methanization process, and the development of advanced methodologies to recover and separate VFAs generated during acidogenesis. Additionally, it requires further development of the conversion efficiency of these VFAs into higher value molecules, especially when using engineered strains, further studies on the thermochemical conversion of generated gases, and standardized methods for biochemical methane potential (BMP) determination and process performance measurements. Finally, it requires a life cycle assessment (LCA) and techno-economic analysis (TEA) to evaluate the environmental impacts, sustainability, and processing costs, along with extensive studies on process scale-up.

This review will therefore provide a comprehensive overview of the necessity to develop a next-generation integrated biorefinery, focusing on the methanization process and particularly on the acidogenesis step to make significant contributions to sustainable waste management and energy production.

## 2. Need for a Better Valorization of Global Waste Streams

According to the International Energy Agency (IAE), sustainable feedstocks for biogas and biomethane production mainly include crop residues, animal manure (from livestock, including cattle, pigs, poultry, and sheep), the organic fraction of municipal solid waste (food and green waste (e.g., leaves and grass), paper and cardboard and wood that is not otherwise utilized (e.g., for composting or recycling), and some industrial waste from the food-processing industry), wastewater sludge, and—for direct production of biomethane via gasification—forestry residues. This assessment considers only those feedstocks that do not compete with food for agricultural land. Biogas and biomethane production in 2018 was around 35 million tons of oil equivalent (Mtoe), only a fraction of the estimated overall potential [32].

**Figure 1:**
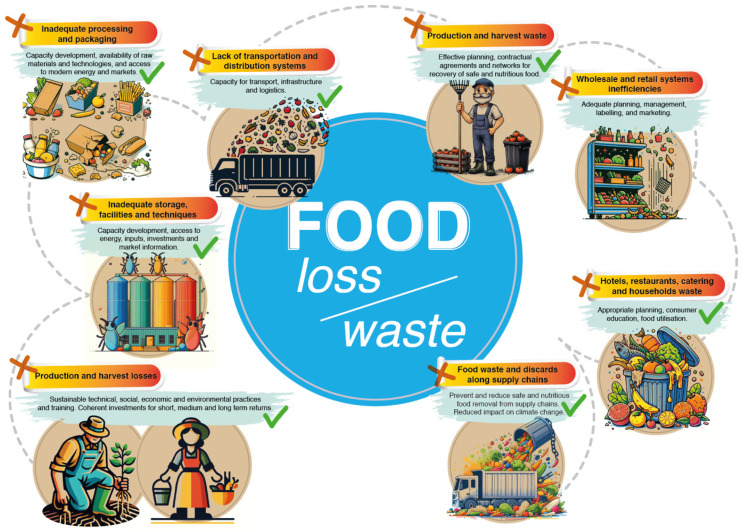
Overview of food loss and waste along the supply chain, illustrating the contributing factors and potential improvements. Adapted from [40].

The annual amount of all waste produced in the world is estimated to be between 7 and 9 billion tons, of which more than 2 billion tons represents municipal solid waste [33]. Figure 1 was adapted from the Food and Agriculture Organization (FAO) of the United Nations (UN). It graphically shows the multiple ways leading to food loss and waste through the different stages of the supply chain. It highlights the major points where inefficiencies and lack of infrastructure contribute to food wastage, beginning with losses in production and harvest due to inadequate storage facilities and techniques, and inadequate processing and packaging. It shows the issues occurring in the transportation and distribution systems, such as limited capacity for transport and logistic infrastructures. The figure also draws attention to wholesale and retail inefficiencies, as well as waste generated by hotels, restaurants, catering services, and households.

According to the UN, ≈13% of all food produced worldwide is lost between the harvesting stage and its arrival at the distribution sites. Additionally, significant amounts are wasted at retail points and during consumption. In total, ≈17% of the global food production is wasted by the households, food services, and retail sectors. In 2022, between 691 and 783 million people worldwide faced hunger, highlighting the urgent need for an improved food distribution and management system. Furthermore, lost or wasted food is responsible for 38% of the total energy consumed within the global food supply chain [34]. Many examples of valorization have been described in the literature for the extraction of valuable compounds from these food waste streams, as well as their conversion into valuable compounds and bioenergy [35,36,37,38,39], among others.

**Figure 2:**
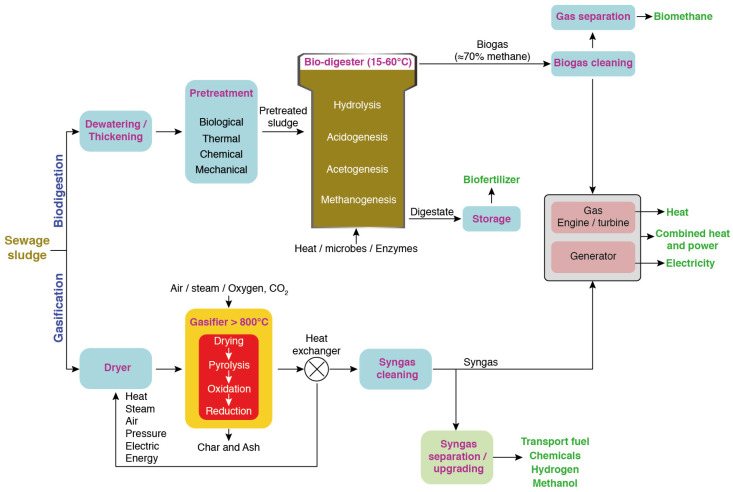
Schematic diagrams of the sewage sludge valorization processes for biogas and syngas production. Adapted from [50].

Sewage sludge constitutes another abundant and inevitable waste stream generated from wastewater treatment plants (WWTPs). In 2017, 45 million tons of dry sewage sludge production was reported worldwide [41]. It represents a semi-solid material containing high organic and inorganic matter that requires processing before disposal [42]. Inadequate wastewater management is associated with many environmental issues, as it contributes to the pollution of the different ecosystems [43,44,45]. At a wastewater treatment facility, the disposal of sewage sludge accounts for ≈50–60% of the total operating costs [46]. Therefore, finding appropriate technologies for its processing not only attenuates its environmental footprint but also allows the conversion of organic content into valuable products [46]. Sewage sludge could therefore be used for the recovery of energy (e.g., biogas, syngas, biodiesel, and bio-oil) and resources (including nutrients, biofertilizers, biochar, heavy metals, and ash for the production of construction materials) [47,48,49]. Anaerobic digestion and gasification of sewage sludge may lead to the generation of biogas and syngas, respectively (Figure 2). These transformation routes were recently well reviewed and covered all of these aspects [23].

It should be noted that from both environmental and economic perspectives, valorizing these waste streams through an integrated biorefinery process is more beneficial than the common current valorization routes mainly involving energy production and soil fertilization. For example, to fulfill the Paris Agreement, many countries started to take measures to reduce and eliminate the emission of greenhouse gases. Knowing that methane and carbon dioxide, the major end-product biogases generated by anaerobic digestion, enhance the greenhouse effect, reducing CO_2_ emissions—even those generated from renewable resources—seems to be a necessity. For instance, in 2022, the rebound in the activity of both passenger and freight transport following the COVID-19 pandemic resulted in a 3% increase in CO_2_ emissions from transport in comparison to the previous year. Furthermore, by analyzing the CO_2_ emission data from 1990 to 2022, it was concluded that transport emissions increased at an average annual rate of 1.7%, faster than any other end-use sector, except for industry (which also grew at ≈1.7%). To reach the ambitious goal of net zero emissions by 2050, the CO_2_ emissions from the transport sector need to decrease by more than 3% annually at least until 2030. This will require the implementation of robust regulations, along with substantial investments to develop the infrastructure for the operation of low- and zero-emission vehicles [51]. As an example, some countries, such as England and Sweden, decided to use electric buses for public transportation instead of biogas buses for a rapid transition to more energy-efficient technologies and fuels [52]. In addition to reducing CO_2_ emissions, the electrification of public transport can also improve air quality and noise pollution [53]. This requires the development of next-generation waste treatment technologies to convert these carbon-rich streams into bio-based chemicals (e.g., VFAs and derivatives) and greener energy, moving beyond mainly producing biogas (e.g., biomethane) and soil fertilizers.

## 3. Optimizing VFA Production for a Next-Generation Methanization Process

**Figure 3:**
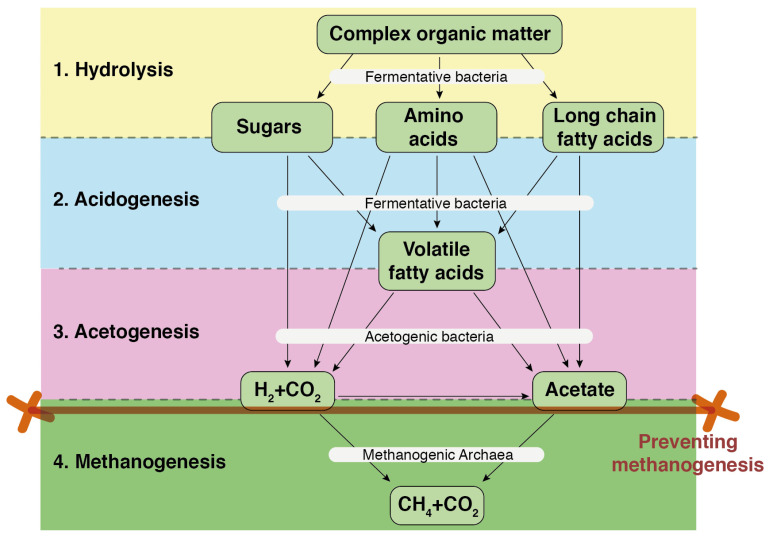
Preventing methanogenesis to enhance the accumulation of volatile fatty acids.

Valorization of key intermediary products, such as VFAs, generated during the anaerobic digestion of waste streams is crucial for designing a next-generation biorefinery. Given the rapid growth rate of acidogenic bacteria (30–40 times faster than methanogens), this stage is typically the fastest in anaerobic digestion. This means that if the reactor is overloaded, carboxylic acids can accumulate, leading to a drop in pH and potentially causing digester failure. To prevent the inhibition of methane production caused by pH reduction, the pH is often controlled by adding external compounds, such as sodium bicarbonate, which raises the pH to neutral values [54]. Efficient recovery of VFAs within the anaerobic digestion process can be achieved by either shortening the reaction time to prevent methanogenesis (Figure 3) or by decoupling the reactional phases, thereby optimizing each stage of methanization. The latter solution appears to be the most appropriate, as it maximizes VFA production and addresses some of the biological and chemical challenges, such as better understanding the mechanisms involving microbial consortia and enhancing microbial resistance to low pH conditions.

VFAs (e.g., acetate, propionate, butyrate, valerate, isobutyrate, and isovalerate, among others) are produced during acidogenesis using monomers generated by the hydrolysis of complex organic matter [1,55,56,57]. Many factors influence the production and accumulation of VFAs, including the composition of the raw material used, configuration of the reactor, pH, temperature, organic load, and hydraulic retention time [58]. It has also been shown that the production yield of VFAs could be enhanced by supplementing the culture medium with electron donors, such as ethanol and lactate [59]. Among the mentioned factors, pH [60,61,62] and temperature [63,64] were extensively investigated. In general, adjusting the pH above 8.0 or below 6.0 enhances the production of VFA and inhibits the growth of methanogens [65]. Some other research works suggested that a medium with an alkaline pH improves the VFA production from anaerobic sludge digestion [66,67], while a pH around 6 was shown to be optimal for VFA synthesis from food waste [55,68]. Regarding the impact of temperature, VFAs are preferentially produced under mesophilic conditions (e.g., 35 °C), whereas high temperatures tend to boost hydrogen production. This is due to the fact that hydrogen-producing micro-organisms (e.g., *Clostridium thermocellum* and *Caldanaerobacter subterraneus*) present higher activity at thermophilic temperatures, which may reach 75 °C [69,70,71,72]. In addition, VFA production seems to be highly influenced by the microbial consortia, as it has been shown that mixed culture fermentation has a significant positive impact compared to monocultures, in addition to the use of non-sterilized medium, thus enhancing the economic viability [73]. Further reading on the factors affecting the production of VFAs, methane, and hydrogen can be found in the literature [74].

VFAs have a wide range of industrial applications. For example, from an environmental point of view, they are important in enhancing the removal of nutrients from effluents, whereas in other fields, such as in material sciences, they could be used, for example, as precursors to synthesize polyhydroxyalkanoates (PHAs), which are considered sustainable substitutes of petro-sourced plastics [75,76]. Additionally, VFAs contribute to the generation of bioenergy due to their use as substrates for hydrogen production [77]. The applications of VFAs have been extensively reviewed in the literature [1,58].

**Figure 4:**
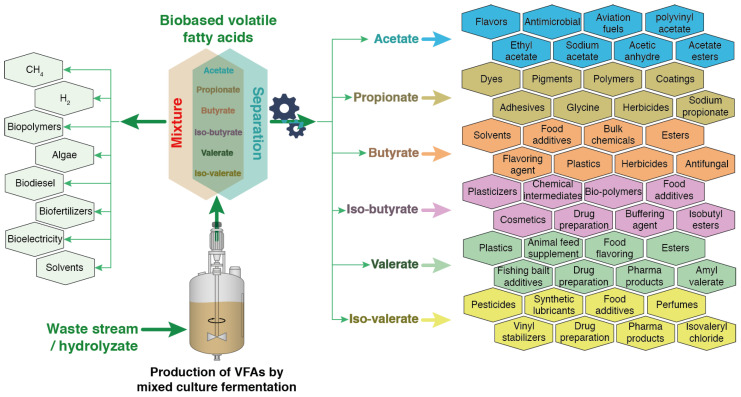
Scope and application of bio-based VFAs as feedstock (as mixture and pure molecules after separation) for the production of multiple commercial products. Adapted from [58].

Figure 4 illustrates a strategic transition in the valorization of bio-based VFAs after their recovery and separation, followed by their subsequent conversion into higher value compounds, compared to their currently preferred usage (i.e., in bioenergy production). This strategy emphasizes not only the economic but also the environmental benefits of such an advanced approach. Separating VFAs enables the synthesis of a wide range of chemicals and materials, including pharmaceuticals, adhesives, and bioplastics, among others, which are of higher economic value than biohydrogen, biomethane, and bioelectricity typically generated from VFAs [74,78]. Diversifying the products derived from VFAs can significantly enhance the economic viability and feasibility of such an integrated biorefinery. Furthermore, this approach concurs with the principles of green chemistry and circular economy by minimizing waste and maximizing resource efficiency [79]. The residual biomass presents an opportunity for energy recovery through anaerobic digestion, thus joining the loop of the bioconversion chain and reinforcing the sustainability of the overall process. This critical analysis will be developed in the subsequent section.

Coming back to the recovery and separation of VFAs, it seems that several scientific and technological barriers need to be overcome for the next-generation valorization process. The process scale-up is facing many challenges due to the numerous limitations associated with the recovery of the produced VFAs [65]. These include, among others, the processing cost, the complexity of fermentation broth composition, issues associated with the recovery of low-concentration VFAs from the fermented stream, and the presence of solid particles that might reduce the process efficiency [80,81,82]. Multiple strategies have been described in the literature to recover VFAs from the fermentation mixture, including liquid–liquid extraction [83], adsorption [84,85], membrane contactors [86], electrodialysis [87,88], and membrane pervaporation [81]. Among these, the utilization of membrane technology for VFA recovery has been highlighted as particularly advantageous in terms of both economic and environmental benefits. The use of this technology for such applications has recently been extensively reviewed [89]. Efficient recovery mechanisms are imperative to the economic feasibility of the process, as they determine the purity and concentration of VFAs, which in turn influence the market value and potential applications of the end products. This process is designed to operate in recycling mode, thereby reducing waste and improving resource efficiency.

## 4. Challenges in the Conversion of VFAs into Valuable Products: A Case Study on Microbial Lipids

**Figure 5:**
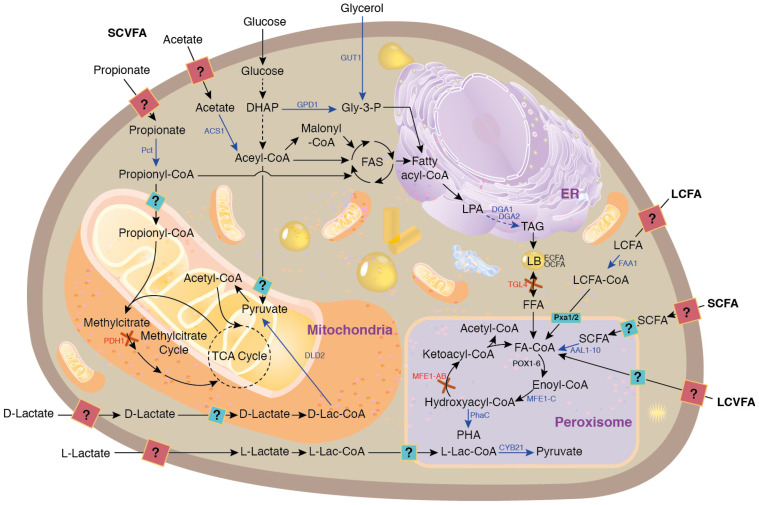
Metabolic pathways by which VFAs are transformed in *Yarrowia lipolytica* into either even-chain fatty acids (ECFAs) or odd-chain fatty acids (OCFAs), as well as polyhydroxyalkanoates (PHAs). Abbreviations: TAG, triacylglycerol; SCVFA, short-chain volatile fatty acids; DGA1/DGA2, acyl-CoA:diacylglycerol acyltransferase; LCVFA, long-chain volatile fatty acids; GPD1, glycerol-3-phosphate dehydrogenase; LPA, lysophosphatidic acid; MFE1, multifunctional enzyme; FFA, free fatty acid; FA-CoA, fatty acyl-CoA; FAA1, acyl-CoA synthase; PHD1, methylcitrate dehydratase; TGL4, triglyceride lipase; GUT1, glycerol kinase; AAL1-12, acyl/aryl-CoA-ligases; ANT1, peroxisomal ATP transporter; PXA1/2, half-ABC peroxisomal transporters; CYB21, peroxisomal lactate dehydrogenase; PhaC, polyhydroxyalkanoate synthase; DLD2, mitochondrial lactate dehydrogenase; FAS, fatty acid synthase; Pct, propionyl-CoA transferase; DHAP, dihydroxyacetone phosphate; Gly-3-phosphate, glycerol-3-phosphate; LB, lipid body. Solid and dashed arrows represent single and multiple steps, respectively. Blue arrows indicate gene overexpression, and red crosses denote gene inactivation. Adapted from [90].

Lipid biosynthesis by oleaginous yeasts is one of the most extensively researched conversion pathways for VFAs. This preference is probably attributed to the predominance of acetate and propionate, which are usually the major VFAs generated during acidogenic fermentation. Odd-chain fatty acids (OCFAs) are synthesized through the incorporation of propionyl-CoA (derived from propionate) in the initial condensation step of fatty acid biosynthesis (Figure 5). Propionyl-CoA is condensed with malonyl-CoA (derived from acetyl-CoA, which in turn is derived from acetate) to form 3-oxovaleryl-ACP, a five-carbon compound that serves as the starting point for OCFA synthesis. Acetyl-CoA also acts as a precursor for the synthesis of even-chain fatty acids (ECFAs). The deletion of the PHD1 gene, encoding the 2-methylcitrate dehydratase in the methyl citrate cycle, prevents the degradation of propionyl-CoA, which promotes the production of OCFAs.

Short- and long-chain fatty acids (SCFAs and LCFAs, respectively), long-chain volatile fatty acids (LCVFAs), and free fatty acids (FFAs) derived from the β-oxidation of lipids can be utilized as precursors for PHA biosynthesis in the peroxisome. Additionally, D- and L-lactate can be metabolized by the cell and serve as precursors of pyruvate, which subsequently enters other biosynthetic pathways. In summary, all these processes utilize VFAs and fatty acids as key precursors, highlighting their critical role in the metabolic pathways for producing valuable biochemical compounds despite the need for further scientific investigations to identify and optimize their membrane transport (Figure 5).

The challenges related to the conversion of VFAs into lipids were extensively reviewed in the literature [27,90]. They include, among others, the toxicity of VFAs beyond a specific concentration and the different assimilation affinities for VFAs by the yeasts. For example, yeast activity can be negatively affected by increasing the concentrations of VFAs, which thus decreases productivity [91,92,93]. The intensity of this effect can vary depending on the yeast strain and the specific type of VFA. Additionally, increased chain length of VFAs is associated with higher inhibitory effects [94,95]. Moreover, increasing the acid concentration in the medium decreases its pH, which may significantly reduce the yeast activity [91,95].

It has been shown that using the salt forms of acetate and propionate—particularly sodium acetate and sodium propionate—has a positive impact on the production of OCFAs by *Y. lipolytica*, probably by reducing VFA toxicity [96]. This approach allowed the use of high precursor concentrations (20 g/L for sodium acetate in this study), which in turn enhanced the conversion rate and led to an increase in the lipid content in the yeast cells. In this study, the authors found that sodium acetate was completely consumed after 96 h of fermentation, while a significant amount of sodium propionate (relative to an initial concentration of 5 g/L) remained even after 168 h of fermentation. This addresses, once again, the need for further research on VFA transport into the cell and the enhancement of various metabolic pathways, as shown in Figure 5. Extensive fundamental research on these aspects may represent one of the many scientific directions to work on over the next decade.

In addition to the above-mentioned challenges, the simultaneous production and conversion of VFAs into valuable compounds show great potential but require more detailed investigations. For example, using mixed cultures (i.e., microbial consortia) for VFA production is generally more efficient than using monocultures; however, the conversion of these VFAs into valuable compounds appears to be more effective when using monocultures.

For instance, in a recent work, the acidogenic fermentation of *Chlorella vulgaris* biomass was conducted in a 1 L reactor at 25 °C, with a hydraulic retention time of 8 days and an organic loading rate of 9 g COD/L/day. Once reaching the steady state, the medium accumulating VFAs was centrifuged to separate the soluble fraction that was then diluted with sterilized water for further conversion into lipids [97]. The results showed that, independently of the VFA concentration, four out of the five tested strains successfully grew in this diluted fraction, thus achieving biomass yields ranging from 0.22 to 0.37 g/g of VFAs. The highest lipid contents were observed for *Cutaneotrichosporon curvatum* and *Cyberlindnera saturnus*, reaching, respectively, 36.9% and 33.9% of dry biomass.

A similar methodology was performed in another work to recover the acidogenic fermentation broth followed by its sterilization and its subsequent conversion by oleaginous yeast [98]. In this work, different concentrations of total VFAs, acid profiles, and carbon-to-nitrogen (C:N) ratios were tested in both real digestate and synthetic media to determine the optimal conditions for lipid production. *Y. lipolytica* demonstrated the ability to grow in all conditions tested, even at high concentrations reaching 15 g/L of total VFAs.

In a more recent work, brewery spent grains were used as substrates for VFA production [99]. The acidogenic fermentation medium was then filter-sterilized (0.2 μm pore size filter) to avoid the precipitation of media components by autoclaving and was used for subsequent conversion into lipids by the oleaginous yeasts *Rhodosporidium toruloides* and *Cryptococcus curvatus*.

These examples and others demonstrate that the conversion of VFAs into lipids by oleaginous yeasts is of high interest but presents some limitations. The acidogenic medium requires sterilization, and the VFA concentrations must be precisely defined before their conversion into lipids by the oleaginous yeasts. Moreover, most of the published studies use non-GMOs for such conversion. Only a few studies have tested GMOs with pure VFAs or their salt forms [96,100,101]. Many questions thus arise regarding the challenges associated with performing a simultaneous acidogenesis and VFA conversion and the limited use of GMOs for such transformations. This is probably due to the difficulties in valorizing the microbial biomass following lipid extraction due to the multiple regulation restrictions.

One of the possible solutions could be the recovery of VFAs during acidogenesis through membrane technology, as previously mentioned. This approach may provide a sterile permeate, which is subsequently processed in a monoculture bioreactor using either a GMO or a native strain. Combining acidogenic fermentation with the extraction of VFAs and their subsequent conversion into valuable compounds could be challenging and therefore requires deeper investigations. For example, to ensure sterility of the VFA extract, membranes with appropriate pore sizes must be used. However, the application of such fine-pored membranes presents the challenge of avoiding fouling, which can negatively impact the system performance and increase processing costs. Thus, while membrane technology is advantageous in producing a sterile extract, it is imperative to find solutions to the fouling issue. This requires a comprehensive study aimed at optimizing the process, potentially testing different membrane materials, or the integration of periodic cleaning steps.

## 5. Integrated Biorefinery Approach for a Next-Generation Methanization Process

An integrated biorefinery approach for a next-generation methanization process aims to maximize the use of available feedstocks and produce greener energy. This includes optimizing methanization through reaction decoupling, recovering and converting VFAs into higher value compounds along with recycling residues, reforming CO_2_ and methane to produce hydrogen, and recovering valuable compounds from the digestate.

The valorization of residual microbial biomass, generated during the conversion of VFAs into valuable compounds (e.g., lipids, bioplastics, etc.), is scarcely studied. This valorization should include the use of both GMOs and native strains. Engineered strains are known for their enhanced efficiency, such as higher yields, and their ability to synthesize unusual molecules, such as OCFAs, compared to wild-type strains. However, the use of GMOs presents significant challenges due to strict regulatory constraints, especially regarding their application for soil fertilization.

A review of the literature indicates that such an integrated biorefinery approach is not sufficiently explored. For example, it has been shown that yeast residue remaining after lipid extraction can serve as an organic source for microbial biodiesel production using *C. curvatus*, thereby enhancing the overall process sustainability [102]. This study highlighted the potential for recycling waste to sustain a continuous bioconversion cycle. The strategy here is quite similar to that of third-generation biomethane production from microalgae. In this process, microalgae serve not only as a biomass source but also as a system for capturing CO_2_. After cultivation, the process includes the recovery of high-value compounds and redirecting the flow of algal residues toward anaerobic digestion.

Residual microbial biomass—particularly that derived from GMOs—generated during VFA conversion can also be gasified to produce syngas (e.g., CO, CO_2_, H_2_, etc.), which is then followed by syngas fermentation or chemical transformation to produce other valuable compounds and biogas (i.e., H_2_ and CH_4_). Despite the extensive research on syngas fermentation, typically using lignocellulosic biomass and waste streams [103,104], scarce information is available in the literature on the gasification of residual microbial biomass. For instance, a recent study demonstrated the feasibility of using syngas generated by the gasification of biogenic residues as a substrate for fermentation to produce short- and medium-chain alcohols using the acetogenic bacterium *Clostridium carboxidivorans* [105]. Syngas production (mainly derived from lignocellulosic biomass) and fermentation for microbial conversion of gaseous substrates to various products have been well reviewed in the literature [106,107,108].

**Figure 6:**
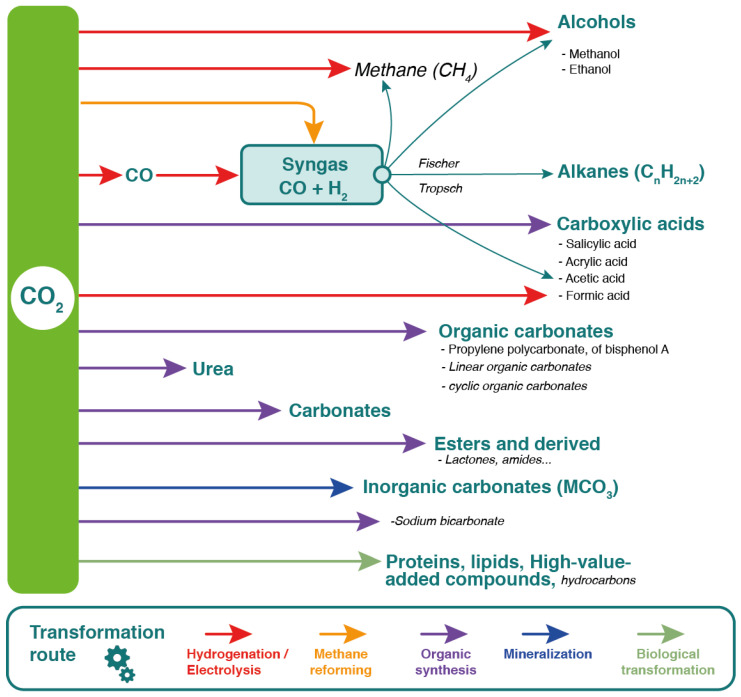
Pathways for CO_2_ utilization and conversion to high-value products. In **bold**: Syntheses carried out at an industrial scale. In *italics*: Syntheses at the “pilot” or “R&D” stage. Adapted from [111].

A sustainable and eco-friendly solution for greener energy production involves CO_2_ reforming of methane to produce syngas (CO + H_2_) [109,110]. The hydrogen produced can be processed and used as biogas for transportation, offering significant environmental and economic benefits. Additionally, various valuable products can be derived from CO_2_ generated by anaerobic digestion. Figure 6 illustrates the pathways for converting CO_2_ through processes such as hydrogenation/electrolysis, methane reforming, organic synthesis, mineralization, and biological transformation into high-value products. Syngas can also be further processed via Fischer–Tropsch synthesis to produce hydrocarbons (e.g., methanol, ethanol), alkanes, and carboxylic acids (e.g., salicylic, acrylic, acetic, formic acids). CO_2_ can also be transformed into organic and inorganic carbonates, esters, urea, and other valuable molecules, highlighting its potential for sustainable production. The technological maturity of these processes ranges from industrial-scale synthesis to pilot and R&D stages.

**Figure 7:**
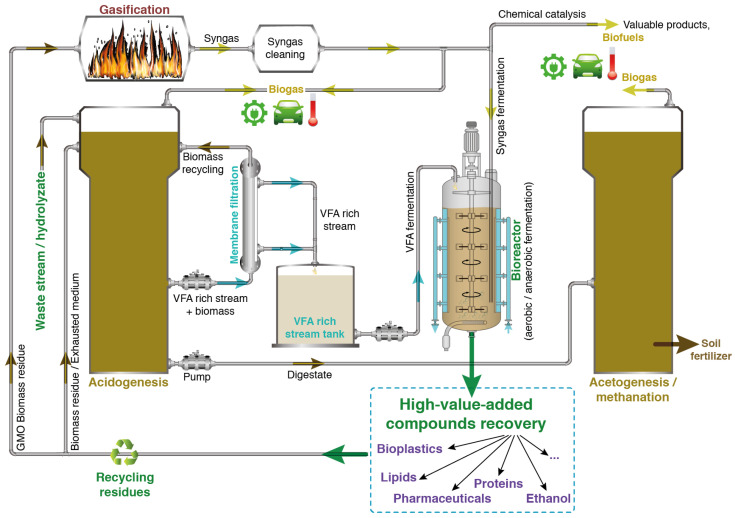
Schematic representation of an integrated biorefinery for a next-generation methanization process providing sustainable solutions for global waste stream valorization.

By analyzing all the above-mentioned aspects, it appears that a next-generation integrated biorefinery for the production of high-value products and bioenergy is feasible despite the presence of numerous challenges in overcoming the numerous scientific and technological barriers. Figure 7 illustrates a suggested integrated biorefinery for a next-generation methanization process, which begins with the introduction of waste/hydrolyzate into the acidogenesis reactor, where VFAs are produced. These VFAs could be continuously extracted through a membrane filtration process, as described above, ensuring a constant outflow toward further valorization. The extracted VFAs serve as substrates (in a mixture or after separation) in either batch or fed-batch fermentation processes, which could be optimized for the production of high-value-added compounds (e.g., lipids, bioplastics, etc.).

After the recovery of these valuable products, the remaining exhausted liquid medium and the residual biomass could be reintroduced into the biorefinery cycle to enhance sustainability and efficiency. Specifically, the non-GMO residues and exhausted liquid medium can be directly recycled back into the acidogenesis step. In contrast, the GMO biomass residues could be directed to gasification, transforming them into syngas. This energy vector can either be used after epuration directly as biogas or converted via syngas fermentation or chemical processes to produce additional high-value compounds and biofuels, as reported above.

The digestate produced could be either used directly or after further stabilization (e.g., by composting) for soil fertilization. It could also be further processed for the recovery of valuable compounds. The biogas generated during gasification and methanization can be used as a heat source for different process units (e.g., pasteurization, fermentation, and gasification), to generate electricity, for transportation, and to produce further valuable compounds.

## 6. Techno-Economic Analysis and Life Cycle Assessment

Integrating a biorefinery approach into the methanization process is essential for sustainable societal development and improved energy efficiency. However, the financial and environmental implications must be evaluated through a techno-economic analysis (TEA) and life cycle assessment (LCA) to facilitate the transition and scale-up.

TEA is a comprehensive approach that combines process simulation with economic evaluation to systematically assess the economic viability of a process. This method effectively identifies the critical bottlenecks and uncertainties that significantly affect the technology’s performance and feasibility. This approach has been applied to the anaerobic digestion process at different stages. For instance, in a recent work, a comprehensive TEA of the anaerobic digestion and the pre- and post-treatment schemes was applied to determine the most feasible pathway for wastewater sludge treatment [112]. The study’s outcomes showed that the scenario with 100% wet oxidation following treatment was the most suitable for the processing parameters and sludge treatment cost. In another recent study, the business case for WWTPs to produce VFAs instead of biogas has been evaluated [113]. The results showed that under favorable conditions, with positive government incentives, future WWTPs could produce high-purity (>98%) propionic acid at USD 3.8 per kg, 35% less than the commercial selling value (USD 6 per kg), which was, in all cases, higher than the current selling price of biogas (USD 0.1 per m^3^). Other research works investigated the thermo-chemical conversion of biogas and syngas, demonstrating energy efficiencies and sustainability [114,115,116].

In addition to TEA, life cycle assessment (LCA, also known as life cycle analysis) is an essential tool to evaluate the environmental impacts of VFAs, their derivatives, and biogas production. This method quantitatively assesses the ecological effects across the entire life cycle, from raw material acquisition to waste management. This allows the identification of the most environment-influencing factors, thus leading to optimization of resource use. The primary objectives of LCA in the context of the current review are to assess the sustainability of using organic waste as feedstock and to compare the environmental impacts of various production technologies. Although so far, no studies have examined the LCA of an integrated biorefinery for a next-generation methanization process, several investigations into VFA and biogas production have highlighted the significant potential of valorizing waste streams for such purposes. For instance, in a recent study, the environmental impacts associated with the bioproduction of VFAs from primary sludge were assessed using LCA methodology to determine the optimal retention time associated with different environmental categories [117]. The results obtained indicated that energy consumption in the different process steps represents a critical challenge for sustainability, as it significantly impacts almost all the evaluated environmental categories. Furthermore, the concentrations obtained suggest that these VFAs could serve as carbon sources to produce PHAs. In a more recent investigation, comprehensive techno-economic and life cycle analyses were performed to evaluate the production of VFAs via anaerobic digestion of food waste and grass. The authors concluded that the environmental impact of VFAs produced using food waste is minimal, presenting a global warming potential ranging between −0.21 and 0.01 CO_2_ eq./kg of product [118].

In addition to conducting LCAs for VFA production, other studies have focused on analyzing the environmental impacts of converting these VFAs into more valuable compounds. For example, in a recent study, LCA was conducted for the production of a fish oil substitute using microalgae from food waste [119]. The results from this study showed that for global warming, terrestrial acidification, freshwater eutrophication, and land use, algae oil involved, respectively, −52 ton CO_2_eq., 3.5 ton SO_2_eq., −94 kg Peq., and 2700 m^2^ eq., per ton of DHA. In comparison, the same impact from fish oil was −15 ton CO_2_eq., 3.9 ton SO_2_eq., −97 kg Peq., and 3200 m^2^ eq. Additionally, compared to canola and linseed oil, algae oil demonstrated a lower climate impact.

LCA of syngas fermentation was also evaluated using a hybrid model for bioethanol production, incorporating co-gasification and fermentation, along with resource recycling and recovery [120]. The authors included in their model the waste heat recovery from hot syngas and power generation from non-converted syngas. The economic feasibility and environmental sustainability of the proposed model were evaluated and compared to traditional first-generation ethanol production. The results obtained suggest that the proposed bioethanol production process is economically viable, with a net present value of USD 18.7 million, an internal rate of return of 13.33%, and a payback period of 6.7 years.

Many studies were performed to assess the feasibility and environmental impact of various similar processes, which are available in the literature for further reading [121,122,123,124,125]. Although the majority of these studies demonstrate the beneficial impact of converting biomass resources and waste streams into valuable compounds and energy, some others have highlighted that while rerouting biowaste from landfills to anaerobic digestion is an effective strategy, it is insufficient to eliminate the emission of greenhouse gases [126]. Moreover, an integrated biorefinery system for a next-generation methanization process should be developed with care, taking into account the variability of feedstocks, the processing costs for the recovery and transformation of valuable compounds, technological barriers, evolving policies, regulations and societal needs, and the numerous scientific mechanisms that need to be understood.

## 7. Future Directions

Many scientific and technological hurdles must be addressed to facilitate the transition toward an integrated biorefinery for a next-generation methanization process, allowing intensive resource use and cleaner energy production compared to the current process mainly used for biogas production and soil fertilization.

Among the scientific barriers is the need for a deeper understanding of VFAs’ metabolism, including both their synthesis and conversion into other valuable compounds. This includes (1) optimizing the acidogenic pathways to enhance the yield of VFAs and selectively to produce specific derived chemicals; (2) improving the assimilation of monomers (derived from the hydrolysis of complex materials) for VFA synthesis (such as identifying specific membrane transporters); (3) increasing the resistance of micro-organisms to acidic conditions to improve the carbon conversion efficiency; (4) identifying the limitations within microbial consortia’s metabolic pathways; and (5) understanding the impacts of emerging micropollutants and pathogenic micro-organisms during digestion. Additionally, the dynamics of microbial communities and their synergy remain not fully understood (antagonistic and/or synergistic effects), requiring further studies to identify the operational parameters and to elucidate how different substrates influence VFA distribution. Moreover, different affinities were observed for the assimilation of these VFAs toward their conversion into higher value products, which requires deeper investigations. In addition, information regarding recycling biomass residues and exhausted medium (especially after VFA conversion) in the digestion or gasification process is scarce. Little is known about the syngas composition of gasified GMOs and their possible conversion via syngas fermentation or chemical processes to produce higher added value compounds and biofuels. Another interesting research field to develop consists of injecting hydrogen (from syngas or produced by electrolysis) into the digester to react with the CO_2_ produced during anaerobic digestion, resulting in the formation of synthetic CH_4_. Overall, in such a system, approximately 60% of the methane produced originates from biomethane (from anaerobic digestion), while 40% is synthetic methane (from methanation).

The technological barriers also pose many challenges. These include cost-effective and eco-friendly technologies for the pre-treatment of feedstocks, most often presenting variable compositions and thus impacting the yields of VFAs. The processing mixtures of VFAs require intensive research work to find solutions for the different issues identified. These include both the recovery of VFAs (e.g., using membrane technology) and separation for individual conversion into higher value compounds. Moreover, membrane fouling, identifying the optimal operating conditions, cost efficiency, and process design for scale-up are among the VFA recovery- and separation-related issues, in addition to the possible formation of water–acid azeotropes, which complicate the separation process. VFAs’ conversion to higher value compounds in a subsequent step (i.e., recovery, separation, and conversion) seems to necessitate deeper investigations. Another scarcely investigated research field is that of biomass recycling (especially that derived from GMO) in either the biodigester (for microbial nutrients’ supplementation) or the gasifier (for syngas production and fermentation, or its chemical transformation) and their related processing parameters to be defined. So far, no study has been conducted to investigate the feasibility of an integrated biorefinery process covering the entire chain from waste stream pre-treatment and hydrolysis, through acidification for VFA production, to the simultaneous recovery and separation of VFAs, their conversion into high-value-added compounds, as well as the recycling of exhausted media and biomass residues back into the system for additional VFA or syngas production, with the ultimate goal of generating biogas and biofertilizers. Furthermore, LCA studies of such an integrated biorefinery system require deeper exploration to ensure environmental and economic sustainability.

The impact of eco-friendly technologies in the integrated valorization chain needs further investigations to cover all the transformation stages from feedstock pre-treatments to biogas/syngas formation and reforming. It is also important to develop knowledge of the positive and negative impacts of methanization on climate, water, air quality, odors, soils, waste, etc., for different deposits and different digestion and energy/agronomic valorization solutions.

## 8. Conclusions

In this critical review, the potential of an integrated biorefinery for a next-generation methanization process was explored, presenting more effective valorization routes for global organic waste streams. Processes involving the biological transformation of waste into VFAs, their subsequent conversion into high-value products, and the recycling of biomass residues, followed by the production of biogas, syngas, biofuels, and fertilizers, are demonstrated to be significantly more beneficial than the currently used methanization process, which mainly focuses on biogas and fertilizer production. Despite numerous scientific and technological challenges associated with the suggested integrated biorefinery process, considerable research has investigated these transformation pathways over the past ten years, with interest expected to grow over the next decade. The development of such an approach will contribute to optimizing the resource recovery, coupled with LCA and TEA to evaluate the environmental and economic impacts. Achieving these objectives will support sustainable development, attenuate the environmental impacts, and allow a rational use of resources.

## Data Availability

Not applicable.
